# User-Centric Proximity Estimation Using Smartphone Radio Fingerprinting

**DOI:** 10.3390/s22155609

**Published:** 2022-07-27

**Authors:** Aleš Švigelj, Andrej Hrovat, Tomaž Javornik

**Affiliations:** 1 Jožef Stefan Institute, Jamova cesta 39, 1000 Ljubljana, Slovenia; andrej.hrovat@ijs.si (A.H.); tomaz.javornik@ijs.si (T.J.); 2 Jožef Stefan International Postgraduate School (IPS), Jamova cesta 39, 1000 Ljubljana, Slovenia

**Keywords:** radio environment fingerprinting, proximity estimation, user-centric, WiFi, BLE

## Abstract

The integration of infectious disease modeling with the data collection process is crucial to reach its maximum potential, and remains a significant research challenge. Ensuring a solid empirical foundation for models used to fill gaps in data and knowledge is of paramount importance. Personal wireless devices, such as smartphones, smartwatches and wireless bracelets, can serve as a means of bridging the gap between empirical data and the mathematical modeling of human contacts and networking. In this paper, we develop, implement, and evaluate concepts and architectures for advanced user-centric proximity estimation based on smartphone radio environment monitoring. We investigate innovative methods for the estimation of proximity, based on a person-radio-environment trace recorded by the smartphone, and define the proximity parameter. For this purpose, we developed a smartphone application and back-end services. The results show that, with the proposed procedure, we can estimate the proximity of two devices in terms of near, medium, and far distance with reasonable accuracy in real-world case scenarios.

## 1. Introduction

Infectious disease models are both concise statements of hypotheses and powerful techniques for creating tools from hypotheses and theories. As such, they have tremendous potential for guiding data collection in experimental and observational studies, leading to more efficient testing of hypotheses and more robust study designs [1,2]. Although person-to-person contact is a major factor in virus spread, recent studies have shown that a person can be infected even after an infected person has left the room [3]. When sharing the same indoor space, close contact can cause viruses to spread via the air, objects, or floor, even after two to three days, if the recommended protective equipment is not used, or disinfection is not carried out [4]. Thus, appropriate monitoring of person-to-person, person-to-place and person-to-object interactions is a crucial issue for post COVID-19 re-opening and for better understanding of disease spread in general [3,5]. Moreover, the spread of communicable diseases in a population is an inherent spatial and temporal process that is of great importance to modern society. For this reason, spatially explicit epidemiological models of infectious diseases are of great importance for improved understanding of the spatial spread of disease through a network of human contacts. Clearly, infectious disease modeling can only reach its full potential if it is more closely integrated with the data collection process, which remains one of the greatest challenges [1]. In particular, ensuring a solid empirical basis for models used to fill data and knowledge gaps is of paramount importance.

Due to the recent pandemic in which social distance has proven to be a critical factor, information about social interactions between users has become essential. Thus, proximity detection is a current research topic. In addition to the disease control domain, proximity detection approaches are also applicable in social sciences to better understand human interaction [6,7], in healthcare where monitoring the number of social interactions could help in the care of patients with certain diseases [8,9], and in the security domain for access control [10], intruder detection [11] and surveillance [12]. Occupancy and proximity detection also have interesting applications in the building control domain for activating heating, ventilation, and air conditioning (HVAC) systems, lighting and other equipment [13,14], emergency management [15], and space utilization and monitoring of occupancy and movement patterns, which are important in pandemic conditions, and more generally in shared workspace environments [16,17,18]. The wide range of applicability of proximity/occupancy detection approaches confirms the need for further research toward non-intrusive, accurate, and energy-efficient solutions.

Various proximity detection technologies have been investigated, such as vision sensors (cameras) [19], infrared [20], audio [21], ultrasonic [22], and radio-frequency-based technologies, such as WiFi [23,24,25,26], Bluetooth low energy (BLE) [27,28,29], RFID [30], and ultra-wideband (UWB) [31]. Most of these approaches (infrared, ultrasound, RFID, audio, cameras) require the deployment of a dedicated infrastructure, which increases the main barrier to entry and, therefore, are not suitable for widespread use. Although some of the methods are very accurate, they can also be very intrusive (audio, cameras) and are usually not well accepted by users. This leaves BLE and WiFi as the two most viable options because wireless infrastructure is extremely widespread, and wireless devices, i.e., smartphones, smartwatches, wireless bracelets, etc., have become ubiquitous personal devices, which makes the approach non-intrusive. In this regard, information and communication technologies (ICTs) can serve as a means of bridging the gap between empirical data and the mathematical modeling of human contacts and networking.

Shortly after the COVID-19 pandemic outbreak, many ad hoc sensor-based technologies emerged for combating COVID-19 [3,32,33,34]. Many of these were based on the use of digital contact tracing solutions [34,35] to evaluate and limit the spread of COVID-19. Various combinations of close-range, proximity-based sensing technologies, such as smartphones, wearables [36], Bluetooth low energy (BLE) beacons [29,34], and positioning-based solutions [37] that use anonymous or randomly coded locations [38], were used. The main goal of the proposed solutions was to identify and inform those who may have been exposed to the COVID-19 virus, so that they could take appropriate actions, such as isolation, care, and treatment [39].

To exploit existing solutions in terms of infrastructure independency, and to pursue the communication trend in which the user is placed at the center, we proposed a novel approach to traffic modeling in [40]. In this approach, the environment is monitored by the user and the infrastructure does not “trace” the user, as occurs with telcos, Internet service providers, Google, etc. In the proposed user-centric approach, the user is completely anonymous and has full control over the data collected by their wireless device(s). It is worth noting, that the user does not need to be connected to any AP/base station to obtain the SSID and RSSI, or any other radio channel parameter. In this way, any user can collect the traces of all radio environment parameters for different networks.

The proposed solution is focused on and exploits existing wireless communication systems consisting of a wireless infrastructure and numerous wireless devices. The wireless communication infrastructure is a complex ecosystem of separate, yet interconnected, systems. It comprises a variety of wireless networks, infrastructure-based or infrastructure-less, both public and private, to which users can connect. Wireless networks consist of multiple base stations/access points connected to the Internet. To be periodically recognized, they transmit their identity and the information required by wireless devices to establish a wireless connection. A common feature of most wireless base stations/access points is that they can be detected by wireless devices without requiring infrastructure intervention. Moreover, in most cases, the infrastructure is not even aware of the wireless devices nearby. A set of detected base stations/access points and estimated radio channel characteristics at a particular location is often called the “radio environment” [41]. The radio waves propagating from the transmitter to the receiver interact with this environment. The interaction is reflected in the received signal as path loss, fading, delay, etc. The radio receivers estimate distortions to improve detection of the transmitted information. If the radio environment is known for all points in a geographical area (i.e., a radio environment map), this information can be applied to locate the user terminal using radio fingerprinting algorithms [42,43,44]. Radio environment maps are obtained through extensive measurement campaigns or by applying calibrated combined deterministic and empirical channel models, which require a perfect knowledge of the geometry and electromagnetic characteristics of the propagation environment. In our research, we use the estimated channel parameters, not to localize the user, but, instead, to estimate the proximity of two users, by exploiting the fact that smartphone terminals that are close to each other have similar or correlated channel distortions.

The main objective of the paper is to propose a user-centric approach that exploits the above-mentioned characteristics of wireless networks to estimate the proximity and, consequently, the “contact intensity“ of a user to other users (or possibly infected individuals). Modern wireless personal devices periodically monitor the radio environment using at least three wireless technologies, i.e., cellular (GSM, USM, LTE), WiFi, and Bluetooth (BT), observing, at minimum, the received signal strength and the identity of all transmitters within range. In the case of WiFi access points (AP), the user monitors the SSID (service set identifier), or MAC address and received signal strength indicator (RSSI), from all the AP in range. Observations of the radio environment can be time-stamped and stored in the wireless device’s memory, thus building a local radio environment trace. When a user wants to determine proximity to an infected person or model their contacts, a user’s radio environment trace is compared to the radio environment traces of others. By delaying the overlap of the traces, it can be determined if the user was still in a room or next to a device (e.g., a coffee maker) later, even though there was no overlap of the traces at that time.

The contribution of the paper is the development of concepts/architectures for advanced user-centric proximity estimation based on radio environment monitoring, and its implementation and evaluation in a testing environment. We define proximity of “person-to-person”, “person-to-place” and “person-to-object”, and investigate innovative methods for the estimation of proximity, based on person-smartphone-recorded radio environment traces. The results show that, with the proposed procedure, we can estimate the proximity of two devices in terms of near, medium and far, with reasonable accuracy in real scenarios.

The remainder of the paper is organized as follows: Related work is discussed in Section 2. In Section 3, we focus on the proposed approach and describe the development of the platform and smartphone application. The proximity estimation procedure is presented in Section 4, while Section 5 evaluates the results for different indoor and outdoor scenarios. In Section 6, we discuss lessons learned, and both the limitations and positive features of the proposed approach. In Section 7, we conclude the paper and identify possible further research directions.

## 2. Related Work

Radio-frequency-based technologies are widely used in research for localization, proximity and occupancy detection, as well as monitoring of movement patterns, since they are widely available and inexpensive while providing data with high accuracy. Important advantages compared to well-known vision and audio solutions include their ability to preserve privacy, and their unobtrusiveness.

Important areas of application include smart buildings, health and safety and security. The application of RFID technology is a mature, widely available and cost-effective solution for tracking, occupancy detection and localization, which has high detection accuracy [45]. Although deployment costs can be relatively high, the technology is frequently used also for indoor locations and sensing solutions to improve the utilization and maintenance of buildings [46]. Due to widely available enabled smartphones, BLE offers a low-cost alternative for occupancy and proximity detection. For instance, in [47], an occupancy estimation approach for indoor environments using BLE technology for emergency management is proposed. The system consists of BLE tags, with a mobile application sending the data to the server site where calculation of building occupancy using different machine learning approaches is performed. Another non-intrusive occupancy monitoring approach, which leverages BLE technologies, is proposed in [48]. In this approach, BLE beacons are programmed to continuously scan the BLE-enabled smartphones in their vicinity. Based on their MAC address and RSSI values, the occupancy and movement patterns are defined. In addition, the approach applies a machine learning model to infer the occupants’ zone-level location. Several studies exist on occupancy prediction for improving facility control and the energy efficiency of buildings, exploiting existing WiFi infrastructure. A solution proposed in [49] utilizes WiFi probe technology to scan active WiFi devices (smartphones) based on a Markov feedback recurrent neural network (M-FRNN) algorithm model for the prediction of occupancy profiles. An occupancy-based HVAC actuation solution, based on existing WiFi infrastructure and enabled WiFi interfaces of occupants’ smartphones, is proposed in [13].

Recently, proximity detection, space occupancy location and tracking have become indispensable for disease control purposes. Especially during the COVID-19 pandemic, contact tracing has became a key measure to reduce virus spread [50]. In [51], the authors showed that mobility patterns were strongly correlated with virus spread. The findings were based on captured daily movement patterns from mobile signals from cell towers in 25 U S. counties. Contact tracing can create an infectious disease transmission network that enables the visualization of actual virus transmission routes, evaluation of outbreak trends, prediction of transmission processes, and development of more effective prevention and control strategies [51]. Proximity/occupancy approaches can be efficiently exploited to count the number of people in a particular area, to identify them and track movement patterns. In [52], the movement patterns of people indoors, based on smartphone WiFi data, was captured. Similarly, the authors in [53] used a WiFi dataset to track the flow of people through buildings. During the pandemic, IoT sensors were used to measure human density, monitor crowd movement, and observe facility usage for crowd monitoring [52]. A mobile crowd monitoring application, which used data from occupancy sensors, cameras, and ticket validation to determine human density in specific areas, was tested in [54].

In [55], the authors proposed an edge-computing prototype to monitor physical distancing that measures the forehead temperature and keeps track of the person count when managing the flow of visitors in public spaces. However, the ad hoc IoT solutions described are unable to interoperate with each other as they are developed using different sensors, data models, communication protocols, and applications without any interoperable means of interconnecting these heterogeneous systems and exchanging data [56]. Thus, the authors in [56] proposed, designed, implemented, and evaluated an interoperable, standard-based, scalable IoT architecture for integrating the disparate Internet of COVID-19 Things (IoCT). The solution contains an effective post-COVID-19 information system for evaluating the transmission risk for both people and places using disparate IoT systems, including proximity-based beacons or global navigation satellite system (GNSS)-based tracking, camera-based COVID-19 risky behavior detection, and contextual indoor geospatial information [56].

Many studies on proximity/occupancy detection have exploited machine learning (ML) approaches on RSSI of BLE and WiFi systems. The Comm2Sense [23] system uses ML to classify the proximity of two devices using the WiFi RSSI, while Virtual Compass, described in [24], in addition to WiFi also includes BLE RSSI values. Additional information exchange (between smartphones) on the distances to known neighbors improved estimated distance. A system involving collaborative processing is presented in [28] which also considers smartphone orientation in proximity detection. The authors of [15] further proposed an improved path-loss model which considers the relative orientation of the smartphones. A comparison between classical modeling approaches and ML when estimating the distance between a transmitter (device) and receiver (device) of BLE signals based on RSSI values is provided in [29]. In [57], the authors present an approach based on fingerprints and Gaussian mixture models to classify the proximity of two devices. Unlike other approaches, the authors use several features, not just a distance metric, to describe the fingerprints collected by the devices. A similar approach was presented in [26], where the authors provided a very detailed analysis of the features used on a much larger dataset.

To leverage and evolve existing solutions and approaches, in the spirit of unobtrusiveness and environmental (home, office, outdoor) independence, without the need for dedicated infrastructure, we have adopted an approach that focuses entirely on the use of radio fingerprints. To create a WiFi or BLE radio fingerprint, all that needs to be collected is information about what devices are available (using MAC address or the SSID of the transmitting device) and what the RSSI value was when the radio environment is scanned by smartphones. Proximity estimation is based on comparing the similarity of the radio fingerprints of different smartphone devices, which means that smartphone terminals that are nearby have similar channel distortions and, consequently, similar radio fingerprints. Thus, users’ smartphones never need to announce their availability. Therefore, the proposed approach is completely independent of the infrastructure as it uses the existing wireless communication systems. In the proposed user-centric approach, the radio environment is monitored by the user, so the user is completely anonymous and has full control over the collected data.

## 3. Concept and Platform Development

**Figure 1:**
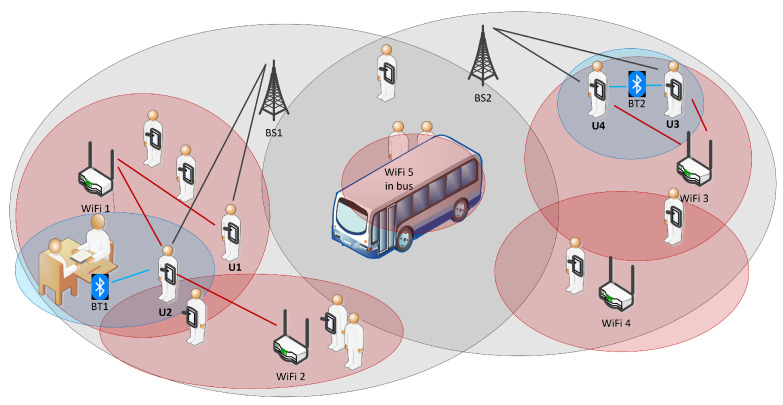
Concept of radio-environment based proximity estimation.

The concept of radio-environment-based proximity estimation is graphically presented in Figure 1. Different users are either in a similar or completely different radio environment. As an example, the radio environment of users U1 and U2 is (BS1;WiFi1) and (BS1;WiFi1,Wifi2;BT1), respectively. There is a certain radio environment overlap; however, as user U1 is not within BT1 and WiFi2, we can assume that the proximity is medium. U3 and U4 exhibit almost the same radio environment, thus the proximity is denoted as close. Note that, if there is no common radio environment, there is no proximity and consequent “contact intensity” (i.e., users U1 and U3) which we denote as far. As people are moving, their radio environment is changing in time. An interesting example is a bus with WiFi5 where the AP is moving with a bus; thus, the users which have the radio environment denoted as WiFi5 at the same time could be in close contact.

The aim of the proposed solution is not only to collect empirical data for mathematical modeling, but also to develop methodologies for practical use of the developed platform, which will be examined by mimicking a real epidemic scenario. In the envisaged real-world scenario, users monitor the radio environment using their devices (e.g., smartphones), but they do not send any data to the server, so they are completely anonymous. Only when a user is identified as infected is anonymized data sent to the server, but only for the period of potential contagion defined by a physician/epidemiologist. The other users are then notified through their devices and can send their anonymized radio environment trace for the same period to the server for evaluation. It is worth noting that the proposed concepts can be used on a smaller scale in local environments (e.g., within a large company, campus, school, etc.) for modeling human contacts and networking in general, and for developing mathematical models and notifying users of potential contagion within the observed local environment. However, in this paper, we focus only on the algorithms for determining the proximity between persons, based on radio fingerprinting.

### 3.1. Monitored Radio Environment

In our research, we limited the wireless technologies to those that can be easily monitored with a smartphone, without any additional features (i.e., an administrative account or additional equipment), so that anyone can run the application. We monitored the following radio technologies and their parameters:

- BLE: Scanner runs for a few seconds and obtains the following information used for fingerprinting:
  - Device MAC (e.g., 08:EF:3B:63:44:A5);
  - Device Name (e.g., LG CM2460);
  - RSSI (e.g., −75 dBm).
- WiFi: Internal Android Wifi ScanResult callback is used and provides the following information used for fingerprinting:
  - Device MAC: (e.g., 38:2c:4a:65:42:d0);
  - SSID: (e.g., K-58);
  - RSSI: (e.g., −48 dBm);
  - Frequency: (e.g., 2417 MHz).
- Mobile (GSM, UMTS, LTE, (5G)): Only radio information of the currently selected technology (e.g., LTE) of the selected mobile operator can be retrieved from the phone. Although this is not particularly useful for proximity detection, we collect mobile cell information for possible further use. Without additional knowledge of the operator’s base station parameters, the information cannot be compared between users, especially if they are connected to different operators.

### 3.2. Smartphone Application and Platform Development

**Figure 2:**
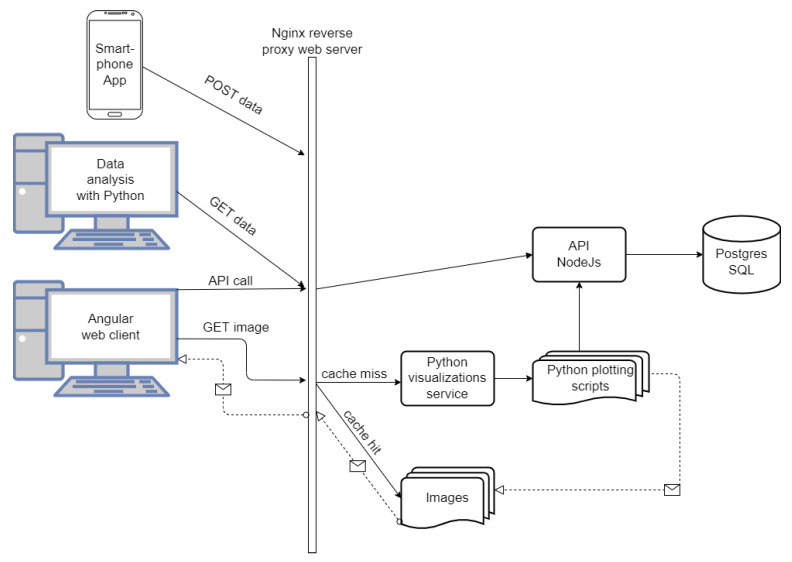
System architecture of testing environment.

For research and testing purposes, we developed a smartphone application that regularly, or on demand, scans the BLE and WiFi networks and also monitors the current mobile network. We consider the environment within a single scan interval as static, assuming that there are small differences in the environment conditions within the scan interval, although the phones are not synchronized. The measurement data is then converted to JSON format and sent to the Postgres SQL database in real-time. The system architecture is shown in Figure 2.

All interactions between the smart application and the back-end are handled via REST API, which is fronted by Nginx. All the operations on data and documentation are also handled through REST API which is implemented by the NodeJS Express [58] program. The data is managed by the Postgres SQL server [59] and interfacing with it is enabled only through the NodeJS server. Postgres was chosen because of its support for JSON storage and full-text search over documentation descriptions. Interaction in NodeJS with Postgres is performed using the Sequelize ORM (Object-Relational Mapper) library [60]. For further processing, data analysis, visualization, and proximity calculation, we use Python scripts that retrieve measurements via REST API. It is important to note that we developed the application only for Android phones from API 28 upwards.

## 4. Proximity Estimation Procedure

With proximity estimation, we want to determine whether a person (smartphone) is near another person (smartphone) or another device (e.g., coffee maker, elevator) that emits a beacon (i.e., BLE) based on the radio fingerprint. In the latter case, we can also define proximity in terms of a time difference (e.g., when two people used the same elevator within a few minutes). It is worth noting that our research does not focus on the possibility of virus transmission between two people, as this is the work of epidemiologists.

We analyzed numerous measurements in different environments, indoor (office and home) and outdoor. In Table 1 there is an example of the radio environment of three phones in different locations in a home environment. A total of 6 WiFi and 3 BLE networks were detected. From the measurements it is quite obvious that Phone 1 and Phone 2 are in the same room, which is the case in this example. The RSSI of two WiFi AP with the best signal (K-58 and K-58-2) are similar, the same is true for two BLE beacons. Phone 3 is obviously in a different location, in a different radio environment.

**Figure 3:**
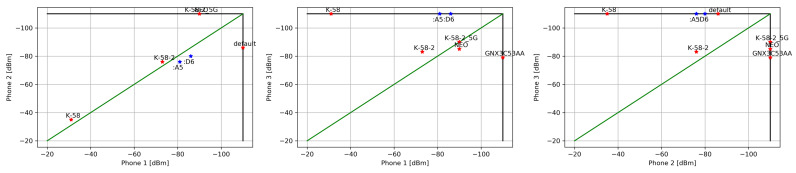
Graphical presentation of phone proximity based on the radio environment. Red stars denote WiFi RSSI measurements, blue stars Bluetooth measurements, and the green line indicates the same signal from both phones. The −110 dBm RSSI level denotes the access points out of the terminal range.

The graphical representation is depicted in Figure 3. The left figure shows the radio environment of Phone 1 (#1) and Phone 2 (#2), while the middle and right figures show the other two combinations. Please note that N/A is shown as −110 dBm for clarity. The WiFi signal is marked in red, while the BLE signal is in blue. The green diagonal line indicates where the same signal is received on both compared phones. In general, measurements closer to the green diagonal line (same RSSI on both phones) and closer to the lower left corner (higher RSSI) indicate close proximity.

Considering the radio propagation properties and preliminary measurements, the following challenges were identified in the development of the proximity algorithm:

- the positions of transmitters are not known (triangulation is not possible);
- the radiated power of transmitters is not known;
- the geometry and the materials of environment (i.e., walls, floors, ceiling) are not known.

The main consequences of the above challenges are that calculating the distance, or the difference in distance, based on the RSSI received from the same transmitter between two devices is not useful. In particular, since small changes in RSSI lead to large changes in distance due to the exponential function, which is especially true for weaker signals (e.g., lower than −85 dBm in the case of WiFi), the method for calculating the range of distance (from $d_{min}$ to $d_{max}$) between two phones (in an empty space) is presented in Equations (1) and (2), respectively.

(1) $$d_{min}=\frac{c}{4\pi f}10^{\frac{P_{t}}{20}}\left|10^{-\frac{P_{r2}}{20}}-10^{-\frac{P_{r1}}{20}}\right|$$

(2) $$d_{max}=\frac{c}{4\pi f}10^{\frac{P_{t}}{20}}\left(10^{-\frac{P_{r2}}{20}}+10^{-\frac{P_{r1}}{20}}\right)$$

where:

$d_{max}$: maximum distance between receivers (rec. at opposite side of trans.) in [m];
$d_{min}$: minimum distance between receivers (rec. at the same side of trans.) in [m];
*c*: speed of light in [m/s];
*f*: transmitting frequency in [Hz];
$P_{t}$: transmitter power in [dBm];
$P_{r1}$: received signal strength at receiver r1 in [dBm];
$P_{r2}$: received signal strength at receiver r2 in [dBm].

**Figure 4:**
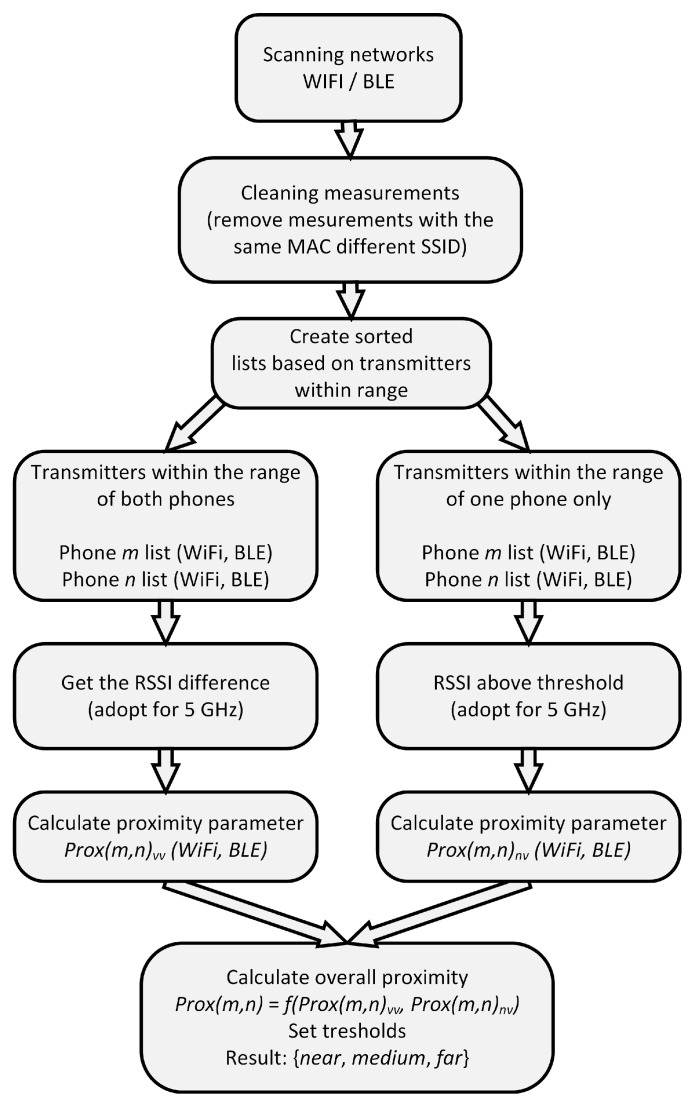
Proximity estimation procedure flow diagram.

As we do not know the positions of the smartphones, the range of distance could be between $d_{min}$ (both phones on the same side) and $d_{max}$ (phones in opposite directions). Another issue is that, even at the same location, there is a small difference (a few dBm) between the RSSI measurements from different phones, particularly from different brands/chipsets used. Therefore, based on measurements, experiments, and heuristics, considering the radio propagation properties, we propose the procedure for proximity calculation presented in Figure 4. The main objective is to define three categories, denoted as:

- near: the phones are close to each other (i.e., few meters, same room);
- medium: the phones are still in the same environment (i.e., same office, neighboring rooms);
- far: the phones are not in the same environment.

In the proposed procedure, we first clean the data by removing duplicate measurements belonging to the same transmitter based on frequency, MAC address and SSID. Next, we create two sorted lists of measurements based on the reception of the signal from the observed transmitters. The first list contains the measurements of the transmitters that are within range of both receivers, and the second list contains the measurements where only one receiver is within the range of the transmitter. If there are no transmitters within the range of both receivers or the signal levels are very low, the proximity is labeled as far. If there are many transmitters within range of both receivers, we consider only the transmitters with the highest signal level and calculate the difference in signal levels. If the signal level is high and the difference is low, and there are also no transmitters with a high signal at one transmitter and not in range of the other, the proximity is labeled as near. More precisely, the proximity parameter Prox(m,n) between Phone (measurement) *m* and *n* is calculated as follows:

(3) $$Prox\left(m,n\right)=w_{vv}Prox{\left(m,n\right)}_{vv}+w_{nv}Prox{\left(m,n\right)}_{nv}$$

(4) $$Prox{\left(m,n\right)}_{vv}=\max\left(0,\sum_{i=1}^{N_{mn}}\left(|RSSI_{m}\left(i\right)-RSSI_{n}\left(i\right)|-w\left(\frac{RSSI_{m}\left(i\right)+RSSI_{n}\left(i\right)}{2}-RSSI_{strong}\right)\right)\right)$$

(5) $$Prox{\left(m,n\right)}_{nv}=\sum_{i=1}^{N_{m}}-RSSI_{m}\left(i\right)+\sum_{i=1}^{N_{n}}-RSSI_{n}\left(i\right)\;\mathrm{if}\;RSSI_{m}\left(i\right)>RSSI_{strong},\;\mathrm{if}\;RSSI_{n}\left(i\right)>RSSI_{strong}$$

where:

Prox(m,n): proximity parameter between phone *m* and *n*;
$Prox{\left(m,n\right)}_{vv}$: proximity calculation for transmitter within range of both receivers;
$Prox{\left(m,n\right)}_{nv}$: proximity calculation for transmitter within range of only one receiver;
$w_{vv};w_{nv}$: weighting factors;
$N_{mn}$: number of all transmitters within range of both receivers/phones;
$N_{m}$: number of all transmitters within range of receiver/phone *m*;
$N_{n}$: number of all transmitters within range of receiver/phone *n*;
$RSSI_{m}\left(i\right)$: received RSSI at receiver *m* from transmitter *i*;
$RSSI_{n}\left(i\right)$: received RSSI at receiver *n* from transmitter *i*;
$RSSI_{strong}$: −70 dBm (WiFi); −90 dBm (BLE);
*w*: the weighting factor (the better the signal, the more relevant the measurement).

In summary, the closer the two transmitters are and the stronger the signal is, the lower the proximity Prox(m,n) is. When the proximity Prox(m,n) is lower than a threshold $T_{1}$, the receivers are classified as near; when it is higher than $T_{2}$, the receivers are classified as far, while all others are classified as medium. The weighting factors and thresholds were defined based on extensive measurements, optimizations, and heuristics for different environments, namely indoor (office/home) and outdoor. First, we performed the optimization process for different indoor environments and found the appropriate thresholds. Then we repeated the process for different outdoor environments. We paid special attention to the determination of the thresholds $T_{1}$ and $T_{2}$, which proved to be most sensitive to the radio environment (indoor vs. outdoor) during the optimization process, while other parameters were not so sensitive. This is understandable because outdoors the signals are weaker, so the difference between thresholds $T_{1}$ and $T_{2}$ must be smaller, which, in turn, reduces the success of the proximity estimation. Please note that special cases like: no received signal at all, only a few very weak signals, etc., are treated separately, resulting in a classification as far, as the above algorithm works well only if there is a sufficient number of transmitters (networks).

## 5. Results

### 5.1. Measurement Campaign

To simplify the labeling process of measurements for development and evaluation purposes, we perform the measurements in a way that adjacent measurements (index or time domain) are labeled as near, those adjacent to a neighbor as medium, while all others are labeled as far in terms of proximity. In this manner, we can easily perform exhaustive measurement campaigns and collect a lot of data for development (i.e., optimization and definition of all weights and thresholds) and evaluation purposes. We performed the measurements in different environments, denoted as indoor (office/home) and outdoor. The significant difference between the two is that, in indoor environments, there are at least some networks with a good signal, while, in outdoor environments, there may be many networks with a very weak signal. First, we calibrated the system and defined the parameters and thresholds for the different environments. The parameters obtained through extensive measurements campaigns for different environments for WiFi and BLE are summarized Table 2.

### 5.2. Evaluation Results

In the first set of results, we present, as an example, the proximities from selected measurements to all other measurements for indoor and outdoor scenarios. The examples presented are selected in a way that shows one with high accuracy and one with low accuracy. We define accuracy as:

(6) $$Accuracy=\frac{Number\_of\_All-Number\_of\_False}{Number\_of\_All}100\%.$$

**Figure 5:**
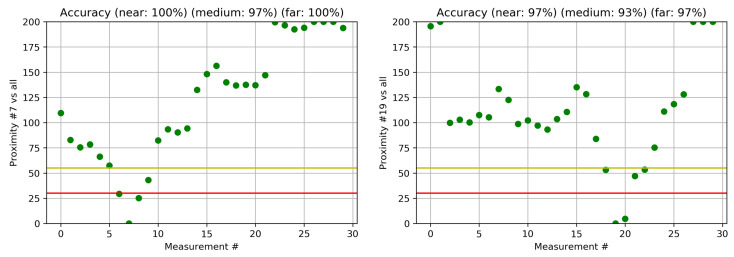
Proximity for indoor measurements. The red and yellow lines are thresholds T1 and T2, and green dots denote proximity to location #7 (**left**) and #19 (**right**).

Figure 5 shows the two examples for the indoor environment. The red and yellow lines denote T1 and T2 thresholds, respectively; thus points below the red line are near, points above the yellow line are far, and points in between are medium. If the proximity is more than 200 or out of range, it is shown as 200. On the left side, we show the proximity of the measurement #7, where the accuracies for near, medium and far are 100%, 97% and 100%, respectively. On the right side, we show an example of the proximity of measurements to measurement #19, where the accuracies are 97%, 93% and 97%, respectively. The accuracy is high due to the large number of spaced-apart transmitters throughout the office buildings.

**Figure 6:**
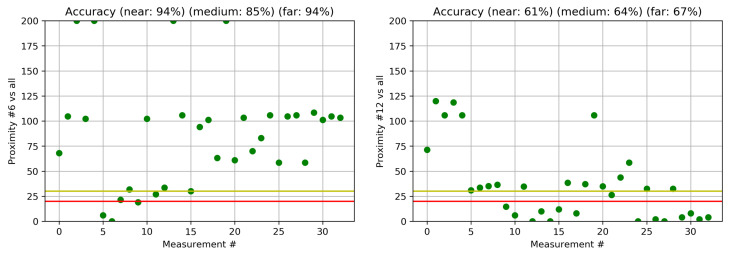
Proximity for outdoor measurements. The red and yellow lines are thresholds T1 and T2, and green dots denote proximity to location #6 (**left**) and #12 (**right**).

Similarly, in Figure 6, we show the results for the outdoor environment. On the left side, we show the results for the proximity of the measurements to measurement #6, with good accuracy (94%, 82% and 94% for near, medium and far, respectively), while on the right side, we show an example of the proximity of the measurements to the measurement #12, where the accuracies are only 61%, 64% and 67% for near, medium and far, respectively. Further investigation shows that, on the left side at point #6, we can see 13 networks, while, at point number #12, we can see only two networks with a weak signal.

**Figure 7:**
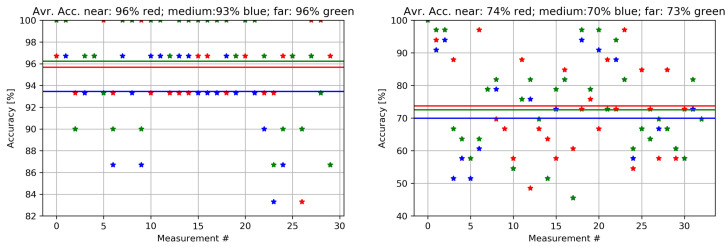
Overall accuracy for indoor (**left**) and outdoor (**right**) real scenario. The red, blue, and green dots denote the single measurement accuracy for near, medium, and far paired devices, respectively, while the red, blue, and green lines illustrate the average accuracy for near, medium, and far paired devices, respectively.

In the second set of results, the accuracy for a set of measurements in two real scenarios is shown in Figure 7. The accuracy of a single measurement is depicted as a red, blue, or green marker for near, medium and far paired devices, respectively. The average accuracy of the experiment is illustrated as straight color lines, namely red, blue, and green for near, medium and far paired devices, respectively. For the indoor environment (left side), the average accuracy for near is 95.8%, while the average accuracy for medium is 93.4%, and the average accuracy for far is 96.2%. For the outdoor environment (right side), the average accuracy for near is 73.7%, while the average accuracy for medium is 70.0%, and the average accuracy for far is 73.4%. As expected, the results for the indoor environments show significantly better results than for the outdoor environment where the accuracy is below 50% for some measurements with only one or two transmitters within range with a weak signal.

## 6. Discussion

During the measurement campaigns, the development of the proposed approach, and development of the system architecture of the testing environment, we encountered several interesting features and limitations, which are discussed below.

The measurement campaigns were chosen to be as realistic as possible, showing only limited areas that proved to be the most challenging. For the larger areas the accuracy was even better, since the individual measurements were farther apart and thus easily recognized as far. We also used the real environments, with the positions of the WiFi and BLE transmitters in their original positions. However, from the results, with respect to “lessons learned”, we can summarize that the accuracy was highly dependent on the distribution of the radio transmitters. Therefore, to achieve significantly better results, it is of the utmost importance to position the transmitters appropriately when deploying WiFi or BLE networks. This means that the positions must not only be optimized for good coverage, but must also be distributed appropriately throughout the building so that the radio fingerprint changes sufficiently between different rooms.To achieve better results the transmitters should not be placed in the center of the building, which is, unfortunately, usually the case, but at the edges. In addition, locations of special interest (e.g., coffee maker, toilet, elevator) can be supplemented with BLE transmitters, installed to improve proximity estimation. By considering the above additional measures, the accuracy can be significantly improved.

In addition to person-to-person proximity estimation, for person-to-object or person-to-place proximity estimation, we must first scan the environment at the particular object, or in the room, and label the radio fingerprint. By comparing the user’s radio fingerprint with the radio fingerprint, we can estimate the proximity using the same procedure, if the user was near an object (e.g., coffee maker ) or within some place (e.g., canteen).

With respect to the mobility of nodes, there are basically two cases: either users move with a node (e.g., on a bus/train with WiFi) or the node is displaced, i.e., moved to a new location. In the first case, users are in the vicinity of moving nodes and within the range of the transmitter, and, due to the metallic structure of the cabin, are isolated from the outside radio environment; consequently, the proximity will be close. In the second case, if the node is displaced or is moving, it may affect the proximity procedure as scanning is not synchronized between users. It may happen that only one user is within the range and another is not, or the signal difference between users is high due to the movement of the node within the scanning period. Since we consider the environment within a single scan interval as static, a moving node may degrade the result, but this also depends on the number, distribution and signal strength of other nodes within the range. The mobility of the node can be detected if there is significant change in the signal level of one transmitter (i.e., the moving one) while the others remain the same.

The battery consumption depends strongly on the Android version used. The reason for this is that the scan functions are implemented differently in the different Android versions but also depend on the battery management of the respective manufacturer. For test purposes, and to collect data for modeling people’s movements, the consumption is not problematic, but, for everyday use, it is necessary to optimize the scanning (e.g., by only performing scanning when the phone is moved). Similar to battery management, measurements are also affected by the model of smartphone or the chipset used. However, since the proposed procedure is robust, small difference in signal strength between smartphones do not significantly affect the outcome of the procedure.

The main limitation of the proposed procedure is the possible lack of transmitters with a good signal in the range. This is often the case in outdoor environments, as we have shown, but, in the home environment (private house) too, there could be only one WiFi and some BLEs. However, the latter is not problematic from an epidemiological point of view, since all members of the household, and even visitors, can be marked as near.

## 7. Conclusions

Smartphones that leverage existing wireless communication infrastructure can serve as a means to bridge the gap between empirical data and the mathematical modeling of human contacts and networking. In this paper, we propose a user-centric approach that exploits the properties of wireless networks to estimate the proximity and, consequently, the “contact intensity“ of a user to other users or places. The main challenges that arise are that the positions of transmitters and their radiated power, and the geometry and the materials of the environment (i.e., walls, floors, ceiling), are not known. To overcome these challenges, we propose a procedure based on radio propagation properties and a heuristic. For the proposed proximity measurement concept, we define the proximity parameter and classify the proximity of two devices in terms of near, medium and far with reasonable accuracy. The results show that the accuracy is better in indoor environments as there are more transmitters with better signals within range.

In future work, based on the proposed proximity estimation procedure, we aim to evaluate networking or contacts between individuals within a single department and within an entire company. In this way, we aim to identify and evaluate individual bubbles or critical points that may have a significant impact on future measures to prevent contacts in the event of an epidemic. Another possible direction is the use of machine learning technologies for proximity estimation. A first step in the machine learning workflow will be to extract features from the raw data that will affect the proximity classification for both sub-environments, namely, the BLE radio environment and the WiFi radio environment. Then the appropriate machine learning algorithm can be selected and evaluated.

## Figures and Tables

**Table 1:** An example of three indoor measurements (home environment).

WiFi			Phone 1	Phone 2	Phone 3
SSID: ‘K-58’	Freq: 2417 MHz	RSSI:	−31 dBm	−35 dBm	N/A
SSID: ‘K-58-2’	Freq: 2417 MHz	RSSI:	−73 dBm	−76 dBm	−83 dBm
SSID: ‘K-58-2 5G’	Freq: 5180 MHz	RSSI:	−90 dBm	N/A	−90 dBm
SSID: ‘NEO’	Freq: 5180 MHz	RSSI:	−90 dBm	N/A	−85 dBm
SSID: ‘default’	Freq: 2437 MHz	RSSI:	N/A	−86 dBm	N/A
SSID: ‘GNX3C5BAA’	Freq: 2437 MHz	RSSI:	N/A	N/A	−79 dBm
**BLE**			**Phone 1**	**Phone 2**	**Phone 3**
MAC: ‘09:28:2C:3A:D3:D6’		RSSI:	−86 dBm	−80 dBm	N/A
MAC: ‘08:EF:3B:63:44:A5’		RSSI:	−81 dBm	−76 dBm	N/A

**Table 2:** Parameters for (WiFi; BLE).

Parameter	Indoor Environment	Outdoor Environment
$T_{1}$	30	20
$T_{2}$	55	30
$w_{vv}$	WiFi → 1/BLE → 1.3	WiFi → 1/BLE → 1.3
$w_{nv}$	WiFi → 0.5/BLE → 0.8	WiFi → 0.5/BLE → 0.8
*w*	WiFi → 0.2/BLE → 0.2	WiFi → 0.2/BLE → 0.2
$RSSI_{strong}$	WiFi → −70/BLE → −90 dBm	WiFi → −70/BLE → −90 dBm

## Data Availability

The data set applied in this study can be obtained on the request sending mail to: ales.svigelj@ijs.si.

## Abbreviations

The following abbreviations are used in this manuscript:

AP	Access Point
API	Application Programming Interface
BLE	Bluetooth Low Energy
HVAC	Heating, Ventilation, and Air Conditioning
JSON	JavaScript Object Notation
MAC	Medium Access Control
ORM	Object-Relational Mapper
REST	Representational State Transfer
RSSI	Received Signal Strength Indicator
SQL	Structured Query Language
SSID	Service Set Identifier
WiFi	Wireless Fidelity
